# Supplementary material for: Targeted nanoconjugate co-delivering siRNA and tyrosine kinase inhibitor to KRAS mutant NSCLC dissociates GAB1-SHP2 post oncogene knockdown
Source: Sci Rep. 2016 Aug 17;6:30245. doi: 10.1038/srep30245 (PMC4987653; doi:10.1038/srep30245)
Supplement: Supplementary Information [file srep30245-s1.pdf]

Targeted nanoconjugate co-delivering siRNA and tyrosine kinase inhibitor to Kras mutant NSCLC reveals Gab1-Shp2 assisted survival pathway post oncogene knockdown

R Srikar,[1] *Dhananjay Suresh,*[2] *Ajit Zambre,*[1] *Kristen Taylor,*[3] *Sarah Chapman,*[4] *Matthew Leevy,*[4] *Anandhi Upendran,*[5,6] *and Raghuraman Kannan*[1,2,7,]*

Departments of [1]Radiology, [2]Bioengineering, [3]Pathology, [5] Medical Pharmacology and Physiology, [6]Institute for Clinical and Translational Science and [7]International Center for Nano/Micro Systems and Nanotechnology, University of Missouri, Columbia, MO 65212, USA
[4]Notre Dame Integrated Imaging Facility, University of Notre Dame, Notre Dame, IN 46556, USA

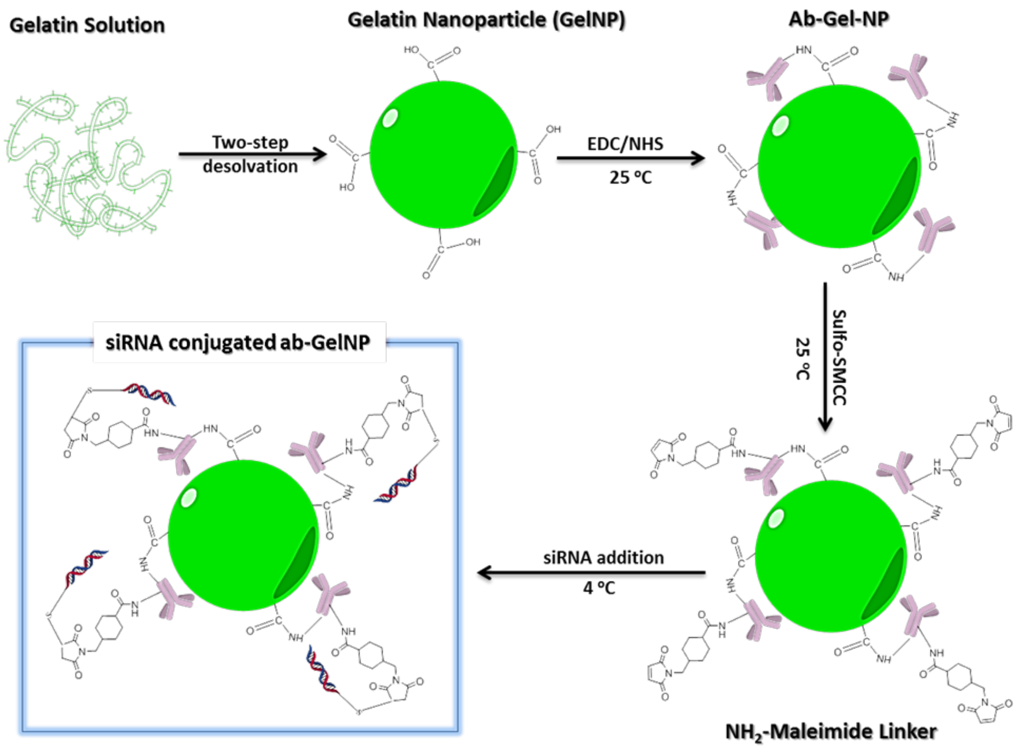

Figure S-1. Synthetic procedure for the preparation of TBN is shown above. Gelatin NP produced by 2-step desolvation process is surface functionalized with cetuximab (Ab) using EDC/NHS reaction to form Ab-Gel NP. The lysine group in Ab is reacted with sulfo-SMCC to generate a reactive maleimide end for conjugating siRNA via Thiol-Maleimide link to form TBN.

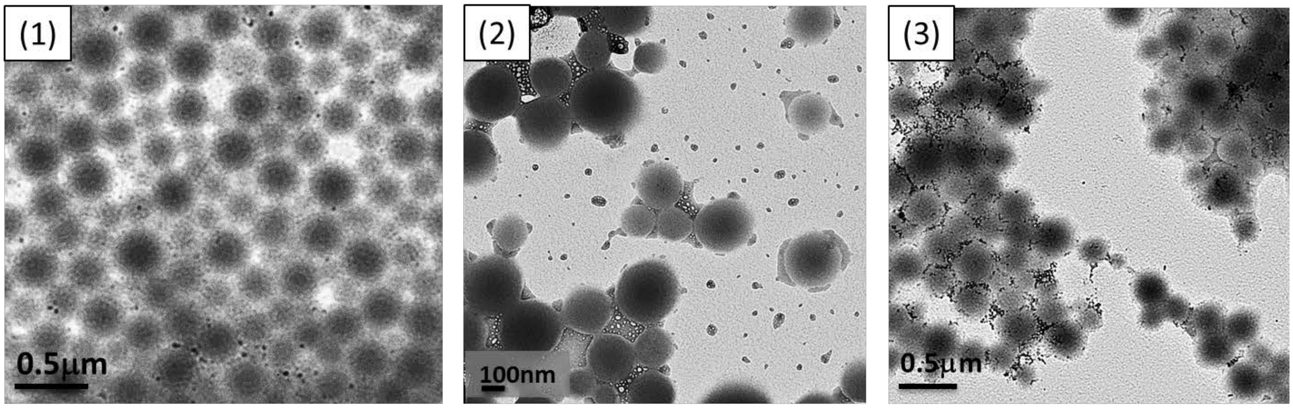

Figure S-2. TEM images of (1) Gelatin NP, (2) Cetuximab functionalized Gelatin NP and (3) TBN. No significant change in the size and shape of the nanoparticles can be observed post surface modifications.

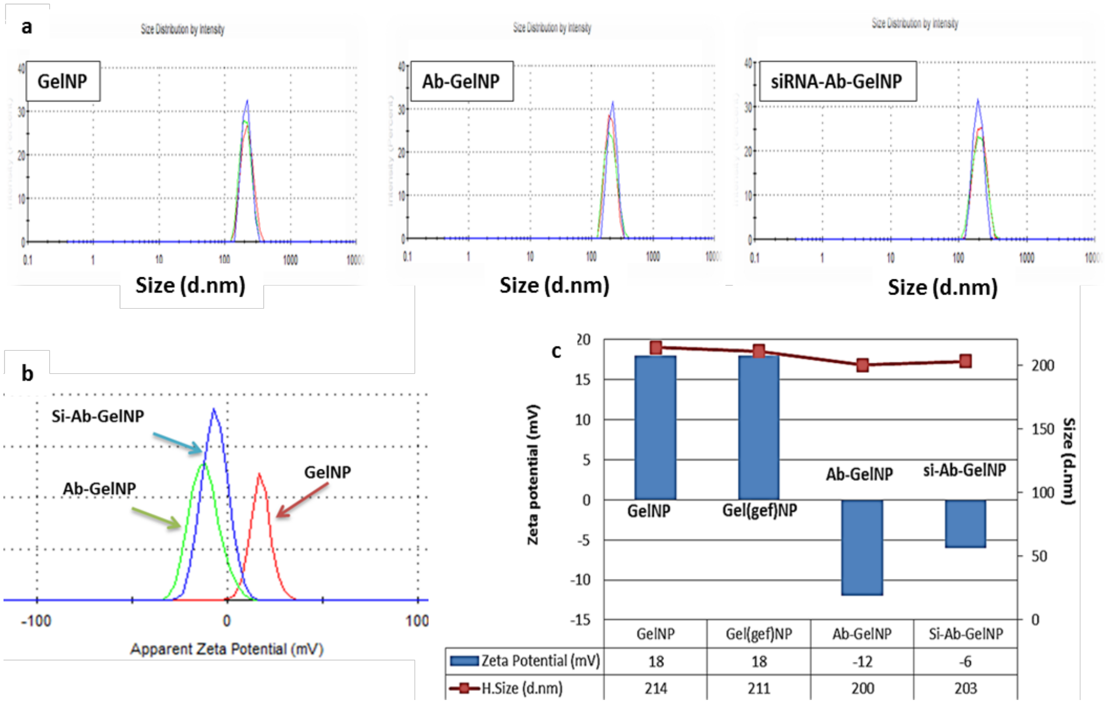

Figure S-3 a) Hydrodynamic size of 225 nm determined through dynamic light scattering method using Malvern Zetasizer Nano ZS. b) Zeta potential of Gelatin NP (Gel NP), Cetuximab functionalized Gelatin NP (Ab-Gel NP) and Ab-siRNA-Gel NP) determined using Malvern Zetasizer Nano ZS. c) Change in Zeta potential upon surface functionalization and siRNA addition is shown above. Incorporation of gefitinib within the core of gelatin nanoparticles does not show any significant change in the zeta potential suggesting the surface properties are maintained before and after gefitinib encapsulation.

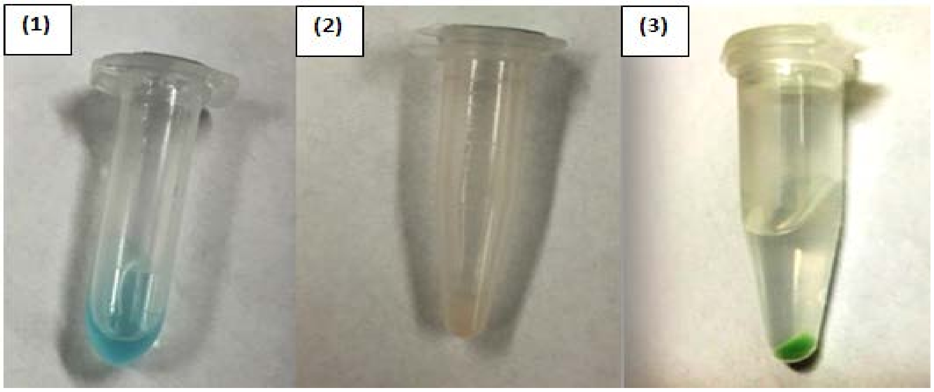

Figure S-4. Visual observation of cy5 conjugated siRNA present in [Ab-GelNP] precipitate. Supernatant shows negligible amount of cy5-siRNA. (1) cy5-siRNA solution, (2) Ab-Gel NP solution and (3) [Ab-GelNP] containing cy5 modified siRNA

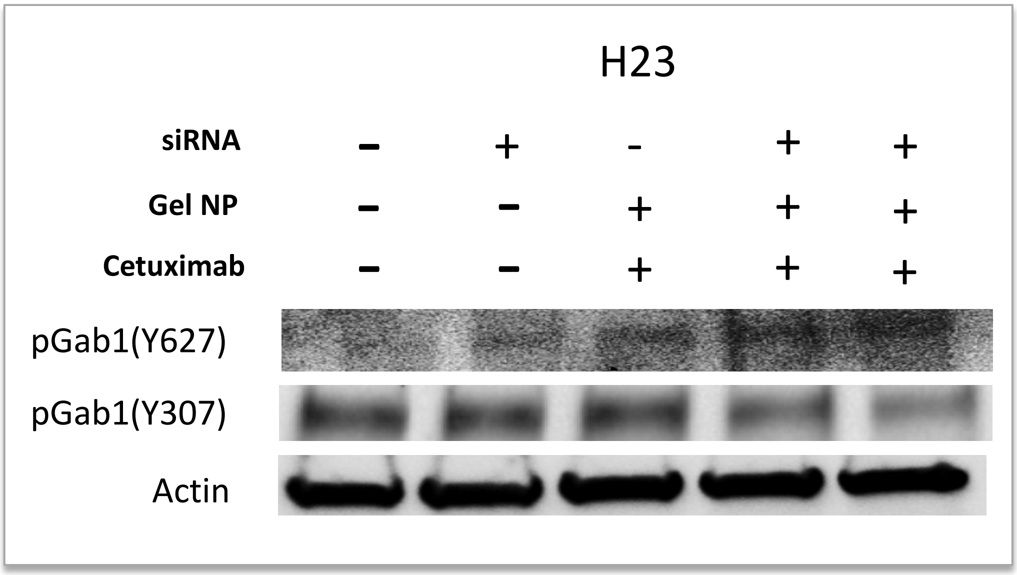

Figure S-5. Western blot analysis of H23 cells incubated with [Ab-siRNA-Gel$_{gef}$] indicates slight increase in the pGab Tyr 627.

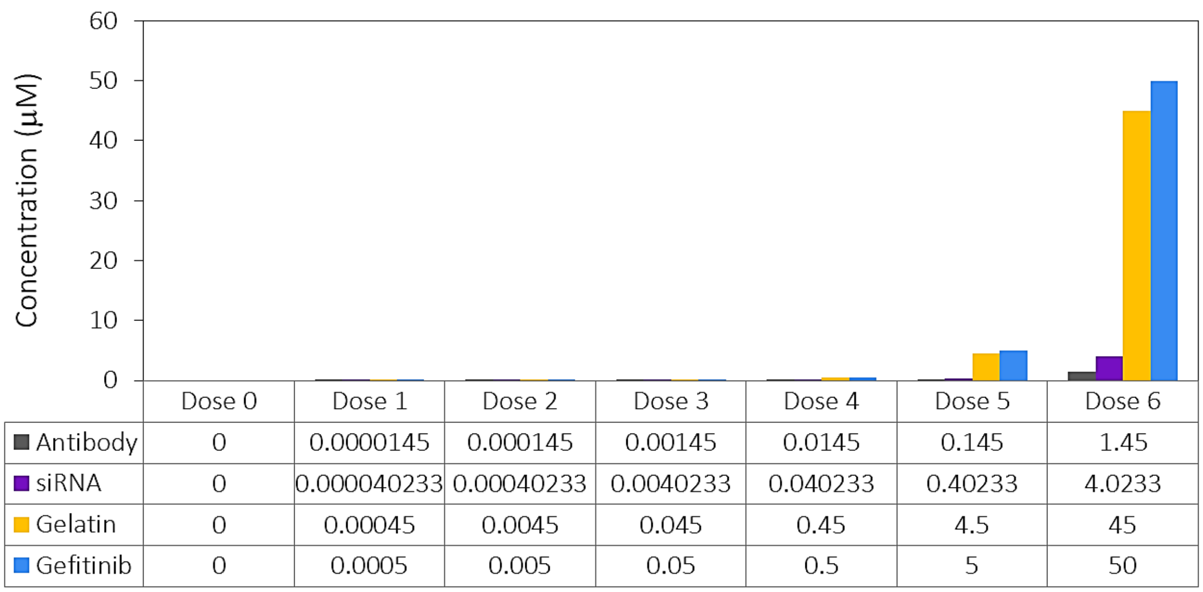

	Dose 0	Dose 1	Dose 2	Dose 3	Dose 4	Dose 5	Dose 6
Antibody	0	0.0000145	0.000145	0.00145	0.0145	0.145	1.45
siRNA	0	0.000040233	0.00040233	0.0040233	0.040233	0.40233	4.0233
Gelatin	0	0.00045	0.0045	0.045	0.45	4.5	45
Gefitinib	0	0.0005	0.005	0.05	0.5	5	50

Figure S-6. MTT dose concentration of nanoparticle and its various analogues used for studying in-vitro toxicity in H23 cells.

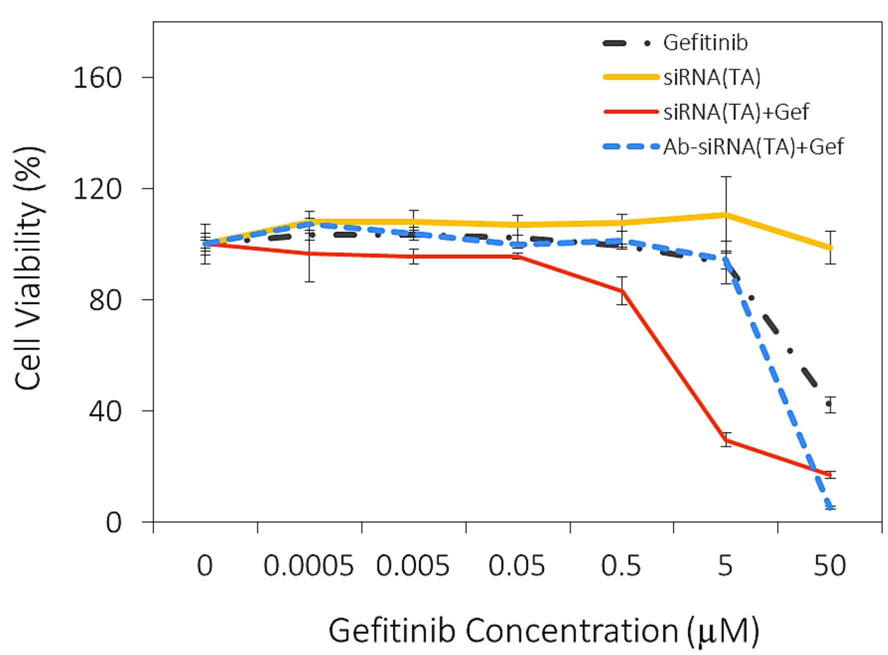

Figure S-7. In vitro cellular viability data of controls such as Gefitinib, siRNA(TA), siRNA(TA)+Gef, Ab-siRNA(TA)+GEF on KRAS mutant H23 cells. Transfected siRNA as such showed no toxicity to the cells. Cells transfected with siRNA and treated with gefitinib exhibited viability of 30% at 5μM gefitinib concentration while Ab-siRNA conjugate upon transfection and treatment with Gefitinib showed cell viability of 5% at 50μM gefitinib concentration. Results indicated post oncogene knockdown, H23 cells gets sensitized towards gefitinib.

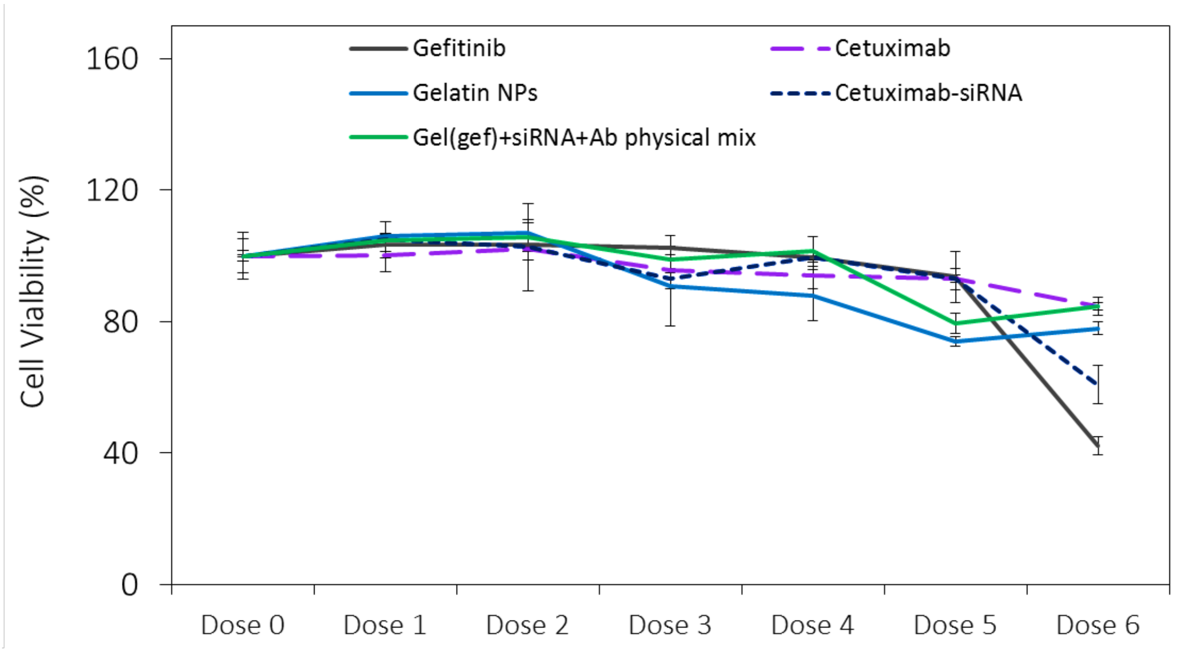

Antibody	0	0.0000145	0.000145	0.00145	0.0145	0.145	1.45
Gelatin	0	0.00045	0.0045	0.045	0.45	4.5	45
Gefitinib	0	0.0005	0.005	0.05	0.5	5	50

Figure S-8. In vitro cellular viability of individual components of TBN and physical mixture of all components together on H23 cells showed minimal cytotoxicity indicating the toxicity caused by TBN in H23 cells is due to the synergistic effect of all compounds present in TBN.

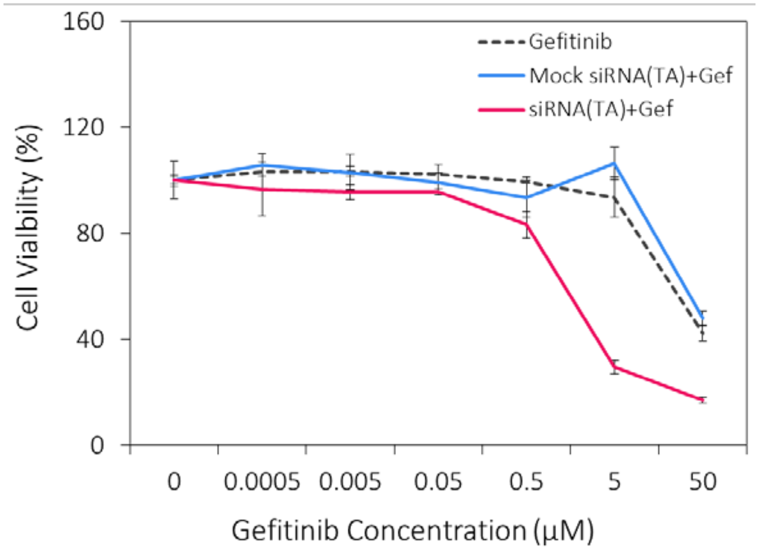

Figure S-9. In vitro cellular viability of transfected mock siRNA followed by treatment with gefitinib on H23 cells. Results indicate no change in viability for mock siRNA compared to the viability of cells treated with gefitinib.

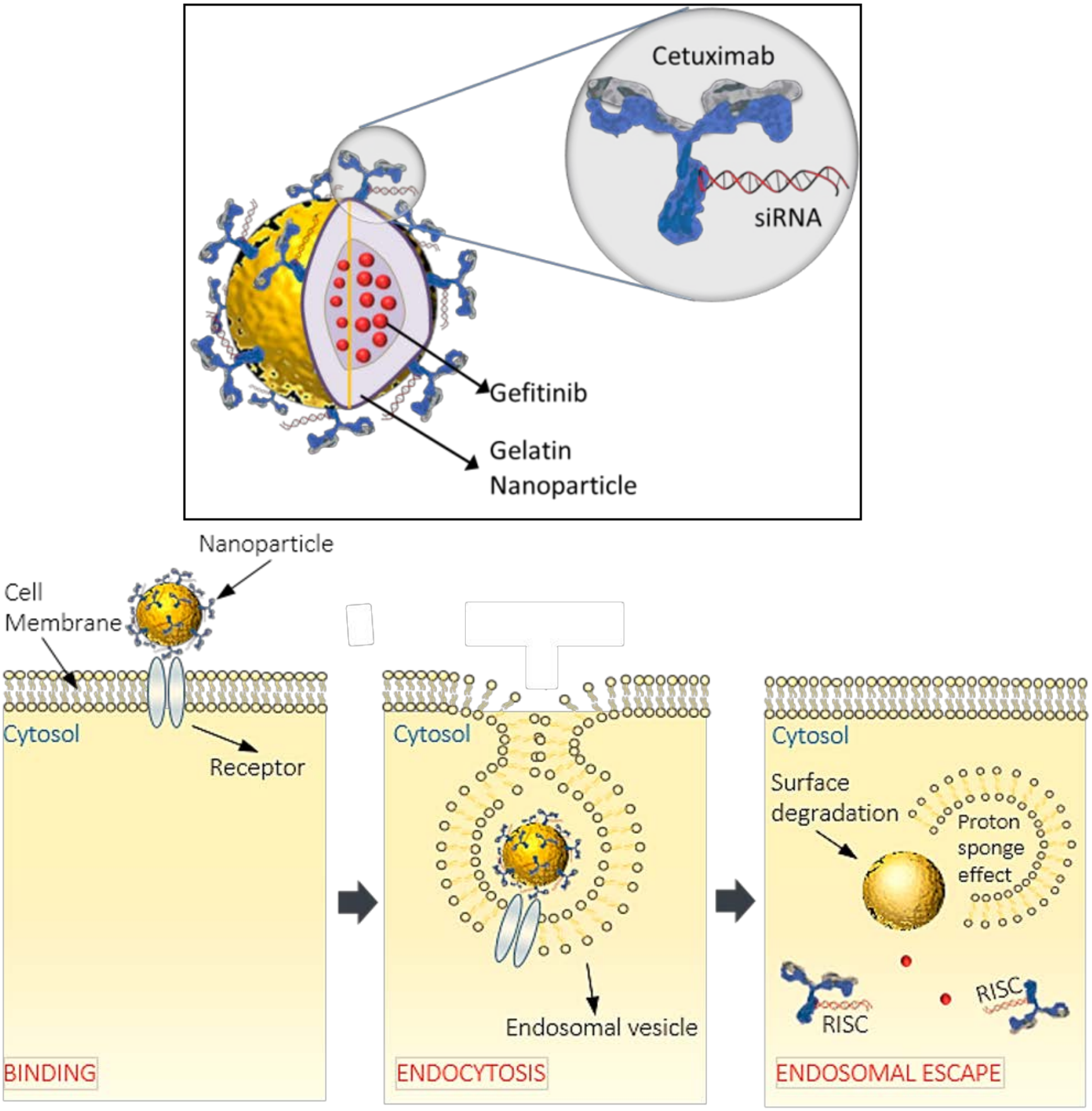

Figure S-10. Mechanism of release of tri-block nanoparticle from endosomes to cytoplasm in H23 cells. The nanoparticle enters the cells using receptor mediated endocytosis and stays in endosomal vehicle. Subsequently, the tri-block nanoparticle release from endosomes possibly due to proton sponge effect and release within cytosol. In cytosol, siRNA forms RISC complex in successfully knocking down KRAS mutant.

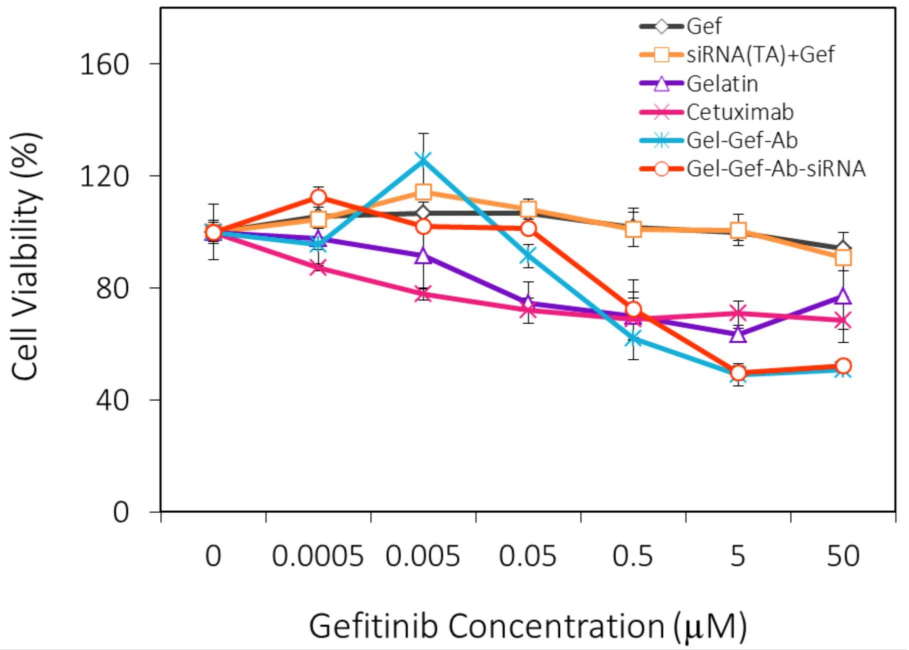

Figure S-11. In vitro cellular viability of TBN and individual components such as Gefitinib, Gelatin NPs, Cetuximab, Gel-Gef-Ab, siRNA(TA)+Gef, Gel-Gef-Ab-siRNA on A549 cells (G12S Mutation).

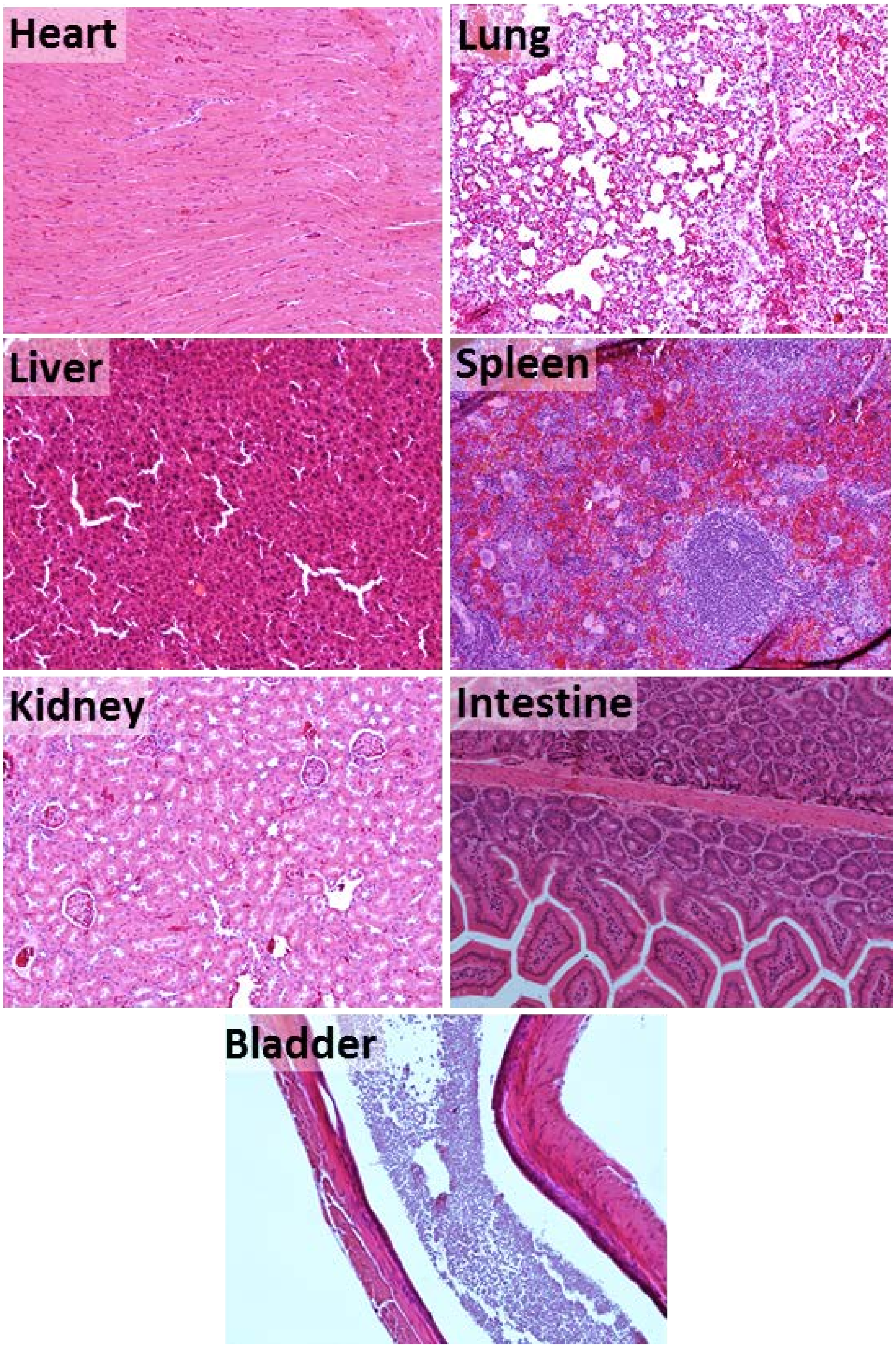

Figure S-12. In vivo histopathological examination of organs upon treatment with large dosage of TBN (80 mg/kg BW) by intravenous administration in normal mice (n=5) for 3 consecutive days.

Table S-1. Concentration of each component comprising the [Ab-siRNA-Gel_{gef}-NP]

Constituent	Mass (µg)	µmol.	Constituent	Absolute Number
**Gelatin**	1000	1	No. of Gelatin NP	**1**
**Gefitinib**	5	1	No. of Gefitinib	**~13500**
**antibody**	50	0.03	No. of Cetuximab	**~350**
**siRNA**	13	0.1	No. of siRNA	**~475**
